# Eco-Friendly Cerium–Cobalt Counter-Doped Bi_2_Se_3_ Nanoparticulate Semiconductor: Synergistic Doping Effect for Enhanced Thermoelectric Generation

**DOI:** 10.3390/nano13202738

**Published:** 2023-10-10

**Authors:** Jamal-Deen Musah, Siu Wing Or, Lingyan Kong, Vellaisamy A. L. Roy, Chi-Man Lawrence Wu

**Affiliations:** 1 Department of Electrical and Electronic Engineering, The Hong Kong Polytechnic University, Hong Kong, China; jamal-deen.musah@polyu.edu.hk; 2 Hong Kong Branch of National Rail Transit Electrification and Automation Engineering Technology Research Center, Hong Kong, China; 3 Department of Materials Science and Engineering, City University of Hong Kong, Hong Kong, China; lingykong6-c@my.cityu.edu.hk (L.K.); lawrence.wu@cityu.edu.hk (C.-M.L.W.); 4 School of Science and Technology, Hong Kong Metropolitan University, Hong Kong, China; vroy@hkmu.edu.hk

**Keywords:** thermoelectric application, energy conversion, nanopartitulate, eco-friendly material, counter–doping

## Abstract

Metal chalcogenides are primarily used for thermoelectric applications due to their enormous potential to convert waste heat into valuable energy. Several studies focused on single or dual aliovalent doping techniques to enhance thermoelectric properties in semiconductor materials; however, these dopants enhance one property while deteriorating others due to the interdependency of these properties or may render the host material toxic. Therefore, a strategic doping approach is vital to harness the full potential of doping to improve the efficiency of thermoelectric generation while restoring the base material eco-friendly. Here, we report a well-designed counter-doped eco-friendly nanomaterial system (~70 nm) using both isovalent (cerium) and aliovalent (cobalt) in a Bi_2_Se_3_ system for enhancing energy conversion efficiency. Substituting cerium for bismuth simultaneously enhances the Seebeck coefficient and electrical conductivity via ionized impurity minimization. The boost in the average electronegativity offered by the self-doped transitional metal cobalt leads to an improvement in the degree of delocalization of the valence electrons. Hence, the new energy state around the Fermi energy serving as electron feed to the conduction band coherently improves the density of the state of conducting electrons. The resulting high power factor and low thermal conductivity contributed to the remarkable improvement in the figure of merit (zT = 0.55) at 473 K for an optimized doping concentration of 0.01 at. %. sample, and a significant nanoparticle size reduction from 400 nm to ~70 nm, making the highly performing materials in this study (Bi2−xCexCo2x3Se3) an excellent thermoelectric generator. The results presented here are higher than several Bi_2_Se_3_-based materials already reported.

## 1. Introduction

The conversion of waste heat to useful electrical energy using thermoelectric (TE) devices has received enormous attention in energy sector due to its efficient energy harvesting technique. The most significant development in thermoelectric research has been obtained in metal chalcogenide materials with a high power factor and considerably low thermal conductivity. High power factor is mainly achieved via simultaneous electrical conductivity enhancement and Seebeck coefficient enhancement [1,2,3]. Although the Seebeck coefficient and electrical conductivity are interrelated, where an increase in one leads to a decrease in the other, several reports have shown that these two parameters can also be enhanced simultaneously [1,2,3]. However, it could not solve the problem of high thermal conductivity, thus affecting the overall TE performance. High electrical transport properties are typically achieved by tuning the complex valence band (E_V_) structure, while the Seebeck coefficient is also improved through band engineering [3,4], nanostructuring [4], and doping [3]. Similarly, efficient phonon scattering approaches, interfacial scattering, and small nanoparticle size-induced scattering reduce the total thermal conductivity. Generally, the thermoelectric conversation efficiency of a material is dependent on the dimensionless figure of merit (zT), described as zT = $\sigma S^{2}T/\kappa_{tot}$ where σ, S, $\kappa_{tot}$, and T are the electrical conductivity, Seebeck coefficient, total thermal conductivity, and absolute temperature, respectively [3,5]. From the zT relation, it is readily seen that efficient enhancement of σ and S while reducing $\kappa_{tot}$ is the goal of every thermoelectric material researcher. Up to date, several TE materials, Bi_2_Te_3_ [6], AgSbTe_2_ [7]_,_ and PbTe [8], Bi_2_Se_3_ [1,5] are recognized to exhibit high TE performance and have attracted intense research. Bi_2_Ch_3_ (where Ch = S, Se, and Te) is attracting extreme attention due to incorporating Bi_2_Te_3_ into commercial thermoelectric generators for room temperature applications. Achieving the innovative potential of TE materials for solid-state generators and refrigerators requires bulk materials with enhanced zT value over unity. While Bi_2_Te_3_ materials are commercialized, Bi_2_Se_3_ is considered a poor TE material due to its low power factor and high thermal conductivity. However, these two materials belong to the same pnictogen chalcogenide group. This has called for attention to bring out the full potential of Bi_2_Se_3_ through several strategies, including doping, band engineering, nanostructuring, etc. Due to this, several dopants (Co, Cu, In, Sb, Te, and rare earth elements) have been reported to contribute immensely to improving the zT of Bi_2_Se_3_.

Elsewhere, we showed that cationic substitutional doping of a single cerium (Ce) dopant for Bi in a typical V_2_VI_3_ (Bi_2_Se_3_) material effectively promotes the simultaneous enhancement of the Seebeck coefficient and electrical conductivity. This leads to improved power factor and enhances thermoelectric performance. By varying the Ce concentration from 0 to 0.4 at. %, improvement in the thermoelectric performance could only reach a zT of 0.24 for Ce_x_Bi_2−x_Se_3_ (x = 0.4 at. %) due to the low σ and hence power factor. The low σ was due to the low carrier concentration. Also, the large particle size led to high thermal conductivity and low TE efficiency. An experimental investigation was carried out by Kadel et al. [9] to explore the TE performance of Bi_2_Se_3_, which showed a zT of 0.096 at 523K. This figure of merit is extremely low and is more than an order of magnitude smaller than that utilized as a desirable TE generator such as Bi_2_Te_3_. The low TE performance in Kadel et al. studies was due to the size-induced increase in the high thermal conductivity compared to recent reports. After this, Sun et al. [10] suggested that the poor performance showed by Bi_2_Se_3_ (zT = 0.096) can be enhanced using copper (Cu) as an interstitial impurity in the framework of Bi_2_Se_3_. The consequence was an enhanced thermal conductivity reduction and improved power factor leading to a hefty zT as high as 0.54 at 590 K. Sun et al. showed that less dopant concentration is useful in enhancing the TE performance of Bi_2_Se_3_. Although a high zT was recorded, the microsized particle for the copper-doped samples significantly increased the thermal conductivity, affecting the overall TE performance. Moreso, Shikin et al. [11] reported a three-dimensional topological insulator of Bi_2_Te_2.4_Se_0.6,_ which exhibited improved TE properties using high-resolution spin and angle-resolved photoemission spectroscopy. While the Fermi level position is determined to be independent of temperature, the spin textures are seen in the Rashba-type valance band surface state and the Dirac-cone state at energies above and below the Dirac point. Also, the observed disorder caused by fractional stoichiometry within the Tellurium/Selenium sublattice does not affect the Dirac-cone state dispersion, leading to an in-plane room temperature Seebeck coefficient of −330 μVK^−1^.

Similarly, using indium and antimony, a dual doping approach has been utilized on Bi_2_Se_3_ [12]. It was evident that the insertion of impurity atoms in the Bi_2_Se_3_ structure led to a crystal lattice distortion and provided significant phonon scattering that favored low thermal conductivity. The low thermal conductivity and the high power factor contributed to the multifold improvement in the figure of merit (zT = 0.47) at 473 K, representing a multifold enhancement. This is among the highest zT for Bi_2_Se_3_ material recorded so far [12]. It has been fundamentally suggested that doping emerges as a prime direction to achieve good TE performance via tuning the intrinsic transport properties, meaning that if a strategic doping approach is well designed, the inherent properties of semiconductors, especially Bi_2_Ch_3_ (Ch = S, Se, and Te), can be harnessed for efficient device performance. Despite the numerous dopants already reported in the literature, from single doping to composite materials synthesis, it is still possible to find novelty from the existing elemental dopants coupled with a suitable design formula to obtain a remarkable TE application.

Here, we report an efficient way to harness high TE performance in an eco-friendly Bi_2_Se_3_ nanoparticulate semiconductor via a counter-doping effect using cobalt (Co) and cerium (Ce), elucidating coupled enhancements in the electrical conductivity and Seebeck coefficient with a drastic decrease in thermal conductivity. We show that despite the self-doping of Co at the interstitial spacing of the Bi_2_Se_3_ crystal lattice, simultaneous improvement of S and σ in an n-type Ce–Co counter-doped Bi_2_Se_3_ nanoparticulate semiconductor fabricated via a single-step solvothermal synthesis route is maintained. Thermoelectric transport measurement, UV-vis spectroscopic analysis, Hall measurement, and density functional theory (DFT) analysis reveal doping-induced enhancement in carrier concentration and effective mass as the Fermi energy approaches the conduction band (E_C_) and more electrons are pumped to the E_C,_ leading to high power factor and zT due to the overlap of the impurity bands, thus narrowing the energy gap. More importantly, the DFT calculation uncovers profound changes to the density of state (DOS) and the band structure of the counter-doped Bi_2_Se_3_ structure, leading to high carrier concentration. Again, the observed simultaneous enhancement in the S and σ at high majority carrier concentration is unusual (Pisarenko relation) [13] and hence differs from that of the bipolar charge carrier transport system. This result aligns with already reported findings substantiating the existing pathway of achieving higher zT in IV–VI compounds and other pnictogen chalcogenides [14]. Theoretical calculations have predicted Bi_2_Se_3_ to demonstrate an excellent thermoelectric property from room to mid-temperature [15,16,17], This, thus, motivates us to experimentally explore the possible material design combination to improve the TE performance practically.

## 2. Experimental Section

### 2.1. Synthesis of Bi_2−x_Ce_x_Co2x3Se_3_ Nanostructures (x = 0, 0.01 0.05, 0.15)

In a typical synthesis of Bi_2−x_Ce_x_Co2x3Se_3_ (BCCS), bismuth (Bi) nitrate pentahydrate (Bi (NO_3_) _3_. 5H_2_O, J&K Scientific Ltd., San Jose, CA, USA, 98%), selenium powder (Se, J&K J&K Scientific Ltd., San Jose, CA, USA, 99%), cobalt (Co) nitrate (Co(NO_3_)_3_, Aldrich, St. Louis, MO, USA, 99.99%), cerium nitrate hexahydrate (Ce (NO_3_) _3_. 6H_2_O, ethanol amine (ACS reagent, Aldrich, St. Louis, MO, USA, 99%), and 2-methoxy ethanol were all purchased and used without any extra purification. In a typical synthesis route, as already explained in our previous studies [1,2], the respective amount of (Bi (NO_3_)_3_. 5H_2_O, Co(NO_3_)_3_, Ce(NO_3_)_3_. 6H_2_O and Se powder were measured and mixed with ethanolamine and 2-methoxy ethanol. Then, as mentioned above, the mixture was stirred continuously for half an hour. The final suspension was then transferred to a 50 mL Teflon-lined autoclave bomb and maintained at 180 °C for a day. After which, the *Bi*_2−*x*_*Ce_x_*${Co}_{\frac{2x}{3}}$*Se_3_* nanostructures were separated from the solvent via centrifugation with DI water and ethanol. The obtained powder is then dried overnight in a vacuum oven.

### 2.2. Characterization of Bi_2−x_Ce_x_Co2x3Se_3_ Nanostructures

The obtained dried powder was subjected to both X-ray diffraction (XRD Bruker SRD –D2 Phaser, Billerica, MA, USA) and UV-vis characterization. The remaining powder was cold pressed into a disc-shaped pellet using a 13 mm die mold and then annealed at 593 K to reduce the residual stress introduced in the pellet during pelleting. The 593 K annealing temperature choice is due to our previously reported optimization [1]. Room temperature Hall measurement, TE property measurement, X-ray photoemission spectroscopy (PHI Model 5802), scanning electron microscopy (FEG-SEM, FEI Quanta 450), and Raman spectroscopy (Renishaw 2000 Raman microscope equipped with a HeNe laser of 633 nm excitation wavelength with laser power of 15 mW) were carried out using the BCCS pellets. Figure S1 (Supporting Information, SI) depicts the elemental composition of the synthesized samples. The TE property measurements, S and σ, are simultaneously measured using ZEM-3, ULVAC. In contrast, the thermal diffusivity, *D*, is measured by the laser flash method (LFA-457, Netzsch, Selb, Germany). At the same time, the Archimedes’ principle and differential scanning calorimetry are used to obtain the density of the pellet and heat capacity (*c_p_*) of the materials. Hence, $\kappa_{tot}$ is calculated as $\kappa_{tot}$ = $\rho \times C_{p}\times D.$ Again, the synthesized BCCS is represented as BS, BCCS-0.01, BCCS-0.05, and BCCS-0.15 for x = 0 at. %, x = 0.01 at. %, x = 0.05 at. %, and x = 0.15 at. %, respectively, for easy referencing. It is important to mention that excess doping above x = 0.15 at. % was not useful as the general TE performance deteriorated as the doping concentration reached x = 0.15 at. %.

## 3. Results and Discussion

**Figure 1:**
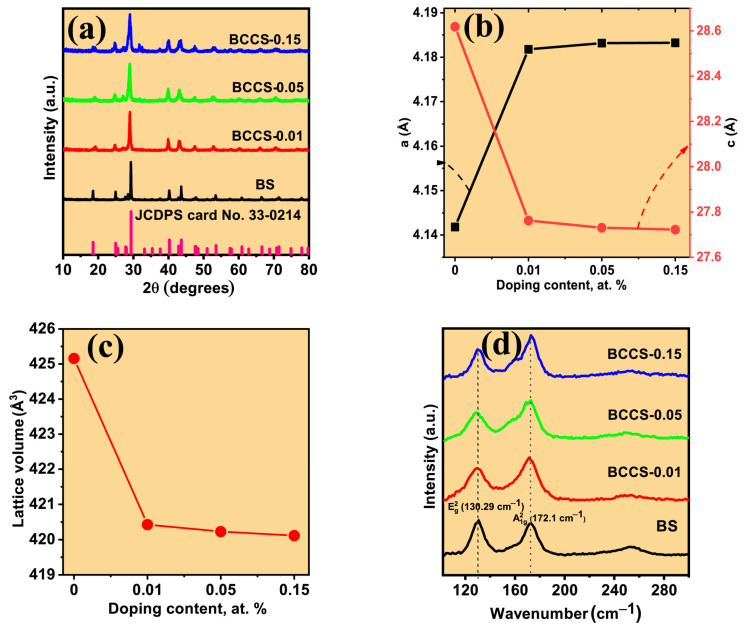
(**a**) The XRD pattern of the synthesized samples: (**b**) calculated lattice parameter; (**c**) lattice volume; (**d**) the Raman spectra of all the BCCS samples.

The phase purity of all the synthesized BCCS counter-doped nanomaterial samples is verified by the X-ray diffraction (XRD) technique. All the measured XRD patterns of pure and Ce–Co counter-doped Bi_2_Se_3_ (Figure 1) match the single-phase standard pattern (JCDPS card No. 330214) of hexagonal Bi_2_Se_3_ material. Upon the increase in Ce–Co impurity content, a noticeable shift of the XRD peaks to a lower 2θ angle signifies a lattice-induced expansion caused by the dopants, where the peak shift saturates for higher doping content. Again, the lattice parameters of all the BCCS samples are displayed in Table S1. With increasing doping content from 0 to 0.15 at. %, the lattice parameters increase from 4.1418 to 4.1832Å while c decreases from 28.6188 to 27.7219 Å, respectively, in which the variation of a and c was ∆a = 0.0414 Å and ∆c = −0.897 Å [18]. This aligns with the literature-reported values for the pristine and doped Bi_2_Se_3_ material [19]. It is revealed that the in-plane lattice parameters of the Bi_2_Se_3_ material increase sharply from BS to BCCS-0.01 and then steadily to BCCS-0.15. This is not the case for the out-plane axis. The decrease in the c-axis causes the entire crystal to shrink, thus decreasing the lattice volume. This represents the formation of a solid solution in the main lattice. It is to be mentioned that the observed decreased lattice parameter is a result of the reduced ionic radius from the host (Bi^3+^) to the dopants (Ce^3+^ and Co^2+^) with no deterioration to the phase stability.

We have estimated the lattice parameters of all the synthesized BCCS nanomaterials using the procedure already used elsewhere [1,2,12], where the c/a ratio decreases with decreasing doping content, suggesting that the adopted doping strategy creates lattice distortion in the host matrix via size reduction effect. Similarly, we numerically estimated the lattice distortion using the approach reported in our earlier studies [12]. It is evident from Table S2 that the introduced dopants not only substitute the host (Bi) or occupy the lattice (Co) but also distort the crystal lattice, contributing to the material’s thermoelectric performance. Similar to our earlier report on indium and antimony dual doped effect in Bi_2_Se_3_ materials [7], the doping-induced lattice distortion enhances phonon scattering, reducing lattice thermal conductivity and improving the material’s zT.

Raman spectroscopy is used to study the vibrational modes of all the synthesized samples to detect the influence of counter-doping in the parent material. The Raman spectra of all the synthesized BCCS samples are depicted in Figure 1d. It is shown that the synthesized samples exhibit exact characteristic peaks belonging to the pristine Bi_2_Se_3,_ $A_{1g}^{2}$, and $E_{g}^{2}$ [12,19]. All the doped samples show almost unchanged Raman spectra, irrespective of the doping content. The lack of Raman shift signifies perfect substitution of the host with the dopants; hence, no significant structural difference is detected (within the detection limit of Raman spectroscopy). The interaction of light with the density of electrons of the chemical bond produces an electromagnetic field in the samples, leading to vibrational and deformation of frequency shift [20,21]. It is noted that none of the Raman peaks in each sample was affected by the doping as manifested in the absence of the wavenumber shift. It is established that the Raman shift is a signature of alteration of bond length caused by external influence, including doping [22]. Ce-Se_2_ and Ce-Se have bond lengths of 2.947 Å [23] and 2.994 Å [24], respectively, while Bi-Se_2_ and Bi-Se exhibit bond lengths of 2.9 Å and 3.04 Å. For a successful substitution of Ce for Bi in a Bi_2_Se_3_ pristine structure, the closeness in the atomic radius of Ce (0.101 nm) [25] and Bi (0.103 nm) [25] coupled with the correspondence in their bond lengths (Bi-Se and Ce-Se) is an indicator of the absence of solid interaction existing between the impurity atom and the host. This, thereby, favors unchanged chemical structure formation. On the other hand, a noticeable broadening of the Raman peaks was observed, which increases with increasing doping amount and indicates the presence of dopants in the pristine structure. Therefore, the Raman characterization of the BCCS samples is in good agreement with the XRD data, suggesting that no impurity phases are formed from introducing the dopants into the pristine Bi_2_Se_3_ material.

Both our previous report (Ce-doped Bi_2_Se_3_) [1] and the current studies (Ce–Co counter-doped Bi_2_Se_3_) show similar behavior to the doping amount, as depicted in the lack of Raman shift as the doping amount increases.

**Figure 2:**
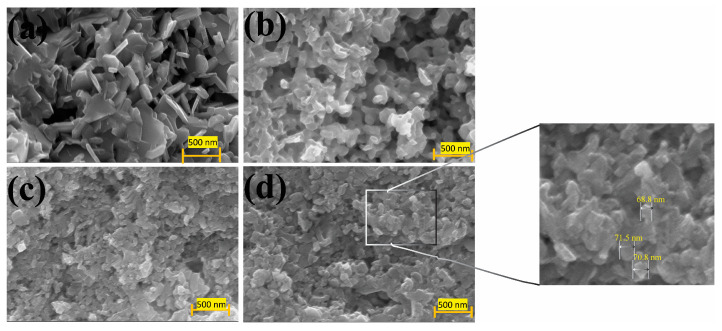
The FEG-SEM morphologies of all the BCCS nanoparticulate samples: (**a**) BS; (**b**) BCCS-0.01; (**c**) BCCS-0.05; (**d**) BCCS-0.15.

We further observed the FEG-SEM images of all the synthesized samples to prove that the TE property enhancement is merely caused by the dopants, where the EDX (Figure S1) and the elemental mapping (Figure S2) are shown. Variation in the particle sizes is observed for the pristine sample (Figure 2a) and the remaining BCCS samples. Although similar particle shapes are found for the pristine (400 nm) and the BCCS samples, the particle sizes decrease as the doping amount increases. Average particle sizes of 95 nm, 88 nm, and 70 nm are found for BCCS-0.01, BCCS-0.05, and BCCS-0.15, respectively. Nanosized pores are also evident in all the synthesized samples (Figure 2). These pores are more significant than tens of nanometers (mean free path of phonons) [26]. This implies that the decrease in the thermal conductivity for the BCCS samples is also contributed by the reduction in the nanosized materials and solid path length and not merely by the porosity. Thus, it is noteworthy that the effect of small particle sizes on the BCCS samples is the increased grain boundaries, which hinder the propagation of lattice vibration, leading to low thermal conductivity. It is, therefore, not surprising why all the BCCS samples demonstrate ultralow total thermal conductivity.

More importantly, the obtained stoichiometries from the EDX analysis are displayed in Table S3. Aside from the decrease in the nanoparticle size with doping, no significant difference in the morphology among the doped samples exists. Similarly, the decline in nanoparticle size with doping (~400 nm to 70 nm), following the increase in the full width at half maximum (FWHM) of the (015) peaks, plays an essential role in thermal conductivity reduction.

**Figure 3:**
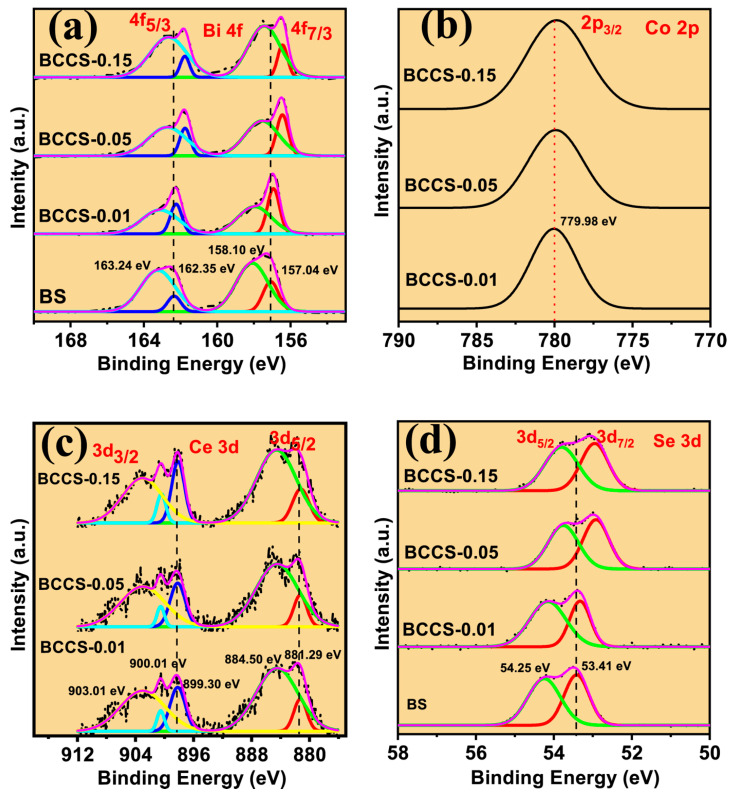
The XPS spectra of the BCCS samples for (**a**) the high-resolution spectra of the Bi 4f core-level, (**b**) the high-resolution scan of the Co 2p spectra, (**c**) the high-resolution scan of the Ce 3d, and (**d**) the high-resolution scan of the Se 3d spectra. (Black dotted lines represent the measured raw data; magenta and black solid lines denote the cumulative fitted spectra; yellow, green, red and blue solid lines represent the deconvoluted spectra).

Determining the oxidation state of all the Bi, Co, Ce, and Se in BCCS is crucial. To obtain those above, we explored the binding energies (BE) of the core levels of the constituent elements (Bi, Ce, Co, and Se) of the BCCS samples and also understand the location of the two dopants (Co and Ce) in the Bi_2_Se_3_ matrix by examining the BE of all the constituent components using the XPS. Figure 3 and Figure S3 depict the high-resolution and full survey scan for the BCCS samples, showing the presence of Bi, Co, Ce, and Se as the most dominant elements in the synthesized samples. The deconvoluted XPS spectra of the Bi 4f, Ce 3d, Co 2p, and Se 3d deduced from the high-resolution spectra for all the BCCS samples are shown in Figure 3. The deconvolution of Bi 4f (Figure 3a) shows two peaks, each with doublet at 157.04 eV and 158.10 eV and 162.35 eV and 163.24 eV, respectively. The spin-orbit energy level spacing of about 5.31 eV is typical for Bi^3+^-based material [12]. From the fitted XPS spectra (Figure 3b), it can be found that with increasing Co^2+^ and Ce^3+^ doping in the Bi_2_Se_3_, the Bi 4f shifts to the low binding energy. The apparent chemical shift indicates a crucial presence of dopants in the pristine materials.

Similarly, for Ce (Figure 3c), binding energies of 881.29 eV and 899.3 eV with a spin-orbit component of 18.01 eV are found, representing Ce^3+^ [1,27]. Table S4 shows the binding energies and intercomponent (spin-orbit) energy separations for the measured Ce 3d peaks in the BCCS samples compared with some reported studies. It is well understood that the Ce 3d core level in the BCCS nanoparticulate exhibits a valence state of 3 (Ce^3+^) with no fingerprint of Ce (IV) compound formation. This implies that the presence of Ce in the Bi_2_Se_3_ substitutes the Bi atoms [1]. The binding energies found at 53.41 eV and 54.25 eV in Figure 3d are well assigned to Se 3d_3/2_ and 3d_5/2_, respectively. A slight shift of the binding energy of the Se 3d to the lower energy is noticed, which increases with increasing doping content. The noticeable change in the BE with doping provides essential information regarding the charge transfer process between the dopants and the Bi_2_Se_3,_ leading to an increase in the electron concentration in the valence band of the Bi_2_Se_3_, supported by the Hall effect characterization.

The Co 2p spectrum in Figure 3b depicts a single peak at 779.98 eV (BCCS-0.01), 779.90 eV (BCCS-0.05), and 779.88 eV (BCCS-0.15), representing the Co^2+^ oxidation state shown for 2p_3/2_ binding energy. This is consistent with experimentally determined reports [28,29]. Elsewhere, Feng et al. [30] reported the binding energies of 778 eV and 793 eV for Co 2p_3/2_ and Co 2p_1/2_, respectively, for Co^3+^ (Co 2p_3/2_) and Co^3+^ (Co 2p_1/2_) whereas the Co 2p peaks positioned at 779 eV and 796.4 eV represents Co^2+^ (Co 2p_3/2_) and Co^2+^ (Co 2p_1/2_). It was found that the peak position for Co shifted to lower binding energy with increasing broadening due to the changes in the local chemical environment. Combining all the characterizations for the BCCS, including the Raman and XPS, the Ce and Co dopants are successfully incorporated into the Bi_2_Se_3_ structure, resulting in the change in the Bi and Se chemical environment, which is pronounced at higher doping content (x = 0.05 and 0.15 at. %).

### Thermoelectric Properties of the Co–Ce Counter Doping in Bi_2_Se_3_ Nanomaterials

Our previous studies of single cerium-doped Bi_2_Se_3_ thermoelectric materials [1] showed the impact of annealing temperature on the thermoelectric performance of pristine bismuth selenide. Then, various concentrations of Ce were chosen as a dopant for the optimized Bi_2_Se_3_ nanostructures, where the effect of Ce doping on the thermoelectric properties of the Bi_2_Se_3_ nanomaterials was discussed. It was evident that the cerium doping simultaneously enhanced σ and *S* while reducing $\kappa_{tot}$. Adopting isovalent substitution for TE enhancement in metal chalcogenides is an established way to decouple the interdependency of σ and *S*. This was shown in our published studies. This is because a host with the same valent as the dopant creates neutral impurities, which oppose less to the conduction electrons. Despite the success in decoupling the Seebeck coefficient and electrical conductivity in the Ce-doped samples, the overall TE performance was low. This was due to the low σ and *S* obtained for the Bi_2−x_Ce_x_Se_3_ samples. A new approach is adopted to modify the synthesized material to improve TE performance further. In this typical method, the Co and Ce counter-doping method is used. As the host’s equal charge dopant (Ce) creates neutral impurities in the host matrix, the Co contributes to the average electronegativity difference (ΔEN), which is crucial to boost the bonding covalency of the parent TE material, arising at the enhancement in the degree of delocalization of the valence electrons. The consequence is improved DOS and promotes ionized defect in the interstitial lattice. Although this, in a way, enhances the carrier concentration, it also reduces the carrier mobility via the impediment of the conduction electron scattering. More significantly, the impurity atoms’ severe dispersion of the phonon vibration leads to reduced total thermal conductivity.

The Ce–Co counter-doped Bi_2_Se_3_ nanostructures were synthesized using a solvothermal synthesis route using the combination of 2-methoxy ethanol and ethanolamine as solvent. The obtained powder was subjected to phase composition analysis and then thorough characterization. All the samples were well indexed to the hexagonal Bi_2_Se_3_ phase, with no prominent impurity phases. This shows that the dopants sit well in the lattice of the host.

**Figure 4:**
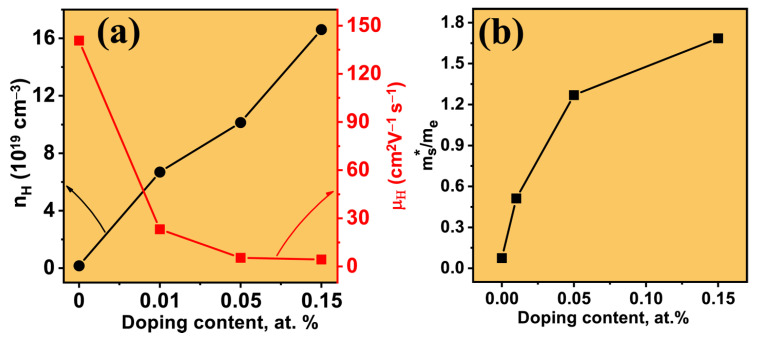
(**a**) The dependence of carrier concentration and mobility on the doping amount and (**b**) doping dependence on the effective mass.

The carrier concentration (*n_H_*) and mobility (*μ_H_*) of all the BCCS samples are measured at room temperature (Figure 4a). Increasing temperature and dopant concentration, as expected [31], increases the *n_H_* from $0.3\times 10^{19}\;{cm}^{-3}\;(x=0\;at.\%)$ to $16.6\times 10^{19}\;{cm}^{-3}\;(x=0.15\;at.\%)$, while the *μ_H_* decreases from 140 cm^2^ V^−1^s^−1^ for the pristine to 4 cm^2^ V^−1^s^−1^ for the highly doped (x = 0.15 at. %) samples. The deterioration of mobility and improvement in *n_H_* with doping content are expected and consistent with the impurity and phonon scattering, respectively. This is because extrinsic scattering from local and extended defects decreases *μ_H_*; hence, the obtained high electrical conductivity with doping indicates improved crystallinity and carrier concentration.

Carrier concentration is hugely affected by the bond strength as well as the EN of the dopants. This implies that using a much lower EN dopant as Ce, compared to Bi, is expected to increase the bandgap. As the introduction of the Co increases the average EN of the dopants, the bandgap reduces. Elsewhere, Kumar et al. [32] showed that the bandgap of a Co_2_MnAl-based alloy increases for a low EN dopant Ge. In contrast, upon doping with a much higher EN dopant (Ga), an increase in the bandgap is observed, depicting that the EN of the doping materials manipulates the optical absorption and the density of state of doped materials [33].

We can describe the transport properties of the BCCS samples by adopting the rigid band approximation, showing that the conduction band structure of the materials does not change appreciably upon doping [34] compared to the pristine. However, experimentally determined properties of the cerium-substituted Bi_2_Se_3_ (*n*$\sim 10^{18}\;{cm}^{-3})$ [1] show inferior electronic properties compared with when Co was further intercalated (*n*$\sim 10^{18}\;{cm}^{-3})$. Considering room temperature conditions, the relationship between the Seebeck-dependent effective mass ($m_{S}^{*}$), S, and carrier concentration is related by Equation (1).

(1) S=2kB2T3eℏ2mS*(1+r)π3nH23

where *K_B_*, *T*, ℏ, $n_{H}$, *e,* and *r* are Boltzmann’s constant, temperature, reduced Planck’s constant, carrier concentration, electron charge, and the scattering parameter (*r* = 0 for acoustic scattering, typical for *T* > 300 K), respectively.

Isovalent doping introduces neutral charge impurities into the crystal lattice of the parent material, and they offer less opposition to conduction electrons than to aliovalent doping. Regardless of the doping type, the counter-doped complex structure like BCCS nanoparticulate material, on the other hand, enhances the disorder in the material, which efficiently scatters phonons and conduction electrons, leading to the possibility of diffusive transport. The degradation of carrier mobility is a hallmark of effective phonon scattering, which results in a considerable decrease in the lattice conductivity. The significant increase in electron–phonon scattering, which reduces the mean free path of conduction electrons, which gets more pronounced with increasing carrier concentration, is thus indicated by the enormous fall in mobility reported for the BCCS samples [35]. On the other hand, due to the significant decline in carrier mobility, it is vital to explore further to understand all possible causes of carrier mobility reduction. Therefore, We have further investigated the scattering mechanisms in the BCCS samples using Equation (S1) and the method already reported elsewhere [2]. We extracted the scattering parameter, *r*, which becomes more pronounced as the doping content increases. It is evident that as the doping content increases, the scattering parameter varies from *r* = −1.5 to *r* = 1.5. This suggests that the most predominant scattering mechanism in the pristine sample is acoustic phonon scattering [36,37]. With the increased doping concentration, a switch from acoustic phonon to pure ionized impurity scattering was observed. This is, therefore, responsible for the massive deterioration of the carrier mobility in the BCCS samples. Similarly, the scattering mechanism in magnetic iron doping in Bi_2_Se_3_, Bi_2_Te_3_, and Sb_2_Te_3_ thermoelectric material is studied elsewhere [38]. It is established that an increase in the doping of the magnetic iron increases the scattering parameter, leading to a change in the electron scattering mechanism from acoustic phonon for the undoped samples to an impurity scattering for the Fe and chromium-doped samples.

All the samples were carefully characterized by the Hall measurement, and using the obtained carrier concentration and the results of the Seebeck coefficient, the $m_{S}^{*}$ is estimated (Figure 4b and Table S5) [39]. Assuming acoustic scattering conditions, the room temperature $m_{S}^{*}$ increases from the pristine ($m_{S}^{*}\;$ = 0.07 m_e_) to the highly doped sample ($m_{S}^{*}\;$ = 1.685 m_e_). The increase in the calculated effective mass with doping indicates higher coupling between the electrons and the lattice, leading to reduced *μ_H_* and thermal conductivity [40]. The estimated $m_{S}^{*}$ values show an ideal way to characterize the band structure changes, primarily upon the subtle changes caused by doping. This implies the system makes the transition from a light mass ($m_{S}^{*}\;$ < 0.1) for carrier concentration not more than 10^18^ cm^−3^ to a much heavier mass ($m_{S}^{*}\;$ > 0.1) for n_H_ of the order of 10^20^ cm^−3^.

We adopted the density functional theory (DFT) using the procedure in our earlier studies to analyze this doping effect on the band structure [1,12,20]. This paper implemented the first principle calculations within the DFT framework. Typically, the DMol^3^ program using Perdew–Burke–Ernzerhof (PBE) of generalize-gradient approximation (GGA) function is adopted to investigate the electronic structures of both Bi_2_Se_3_ and Bi2−xCexCo2x3Se3 material with a 2 × 2 × 1 supercell utilizing a plane wave kinetic energy cutoff of 500 eV. Again, the DFT Semi-core Pseudopots (DSPP) core treatment was chosen to replace the core electrons with a single effective potential [41]. More notably, the k-point of the Brillion zone was maintained as 3 × 3 × 1 for the geometry optimization and 6 × 6 × 3 for high-quality electronic structure calculation.

**Figure 5:**
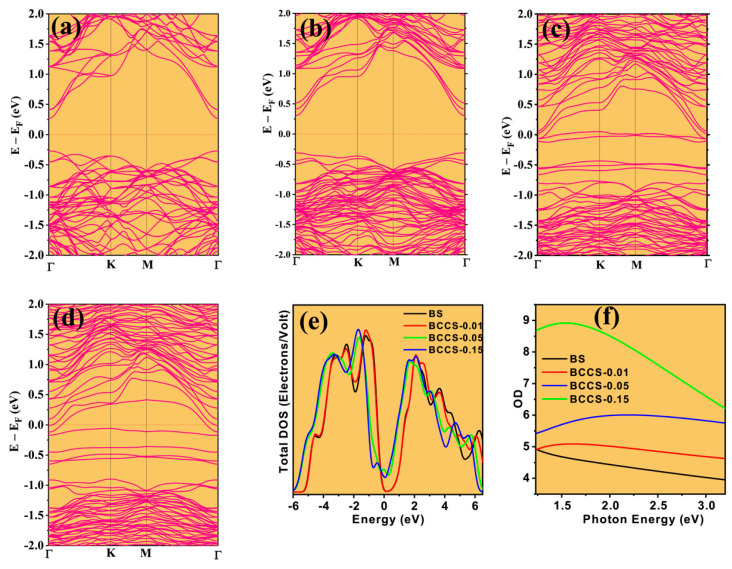
The calculated band structures for the (**a**) pristine and the (**b**–**d**) Ce–Co counter-doped samples and (**e**) the calculated total density of state and (**f**) optical density of state for the pristine and counter-doped samples.

The sensitivity of the pristine material to the Co and Ce counter-doping is shown in the changes to the band features (Figure 5a–d). More so, our calculated band structures for the pristine and all the doped samples elucidate the evolution of new bands near the Fermi level, which is associated with the transfer of electrons from the impurity atoms to the host. Although the band gap decreases with doping, incorporating extra bands near the Fermi level further enhances the electrical conductivity. Again, the calculated total density of state (TDOS) for all the BCCS samples is shown in Figure 5e. The valence band shifts downwards by 0.9 eV for the highly doped samples (x = 0.05 and 0.15 at. %), while that of the x = 0 and x = 0.01 at. % remains unchanged. 

Similarly, the conduction band is slightly occupied with the counter-doped atomic-related bands, crossing the Fermi level. This behavior is responsible for the samples’ n-type nature; hence, the Dirac point is expected to be lowered in energy. Although our calculations could not stoichiometrically represent the experimental data (for x = 0.05 at. % due to the crystal structure’s bulkiness), the cobalt and cerium counter-doping effect in the Bi_2_Se_3_ nanoparticulate semiconductor material is explicitly shown at extremely low and higher doping content.

We used the UV-vis spectroscopy analysis to estimate the synthesized samples’ optical density (OD), shown in Figure 5f. The OD increases with doping content with decreasing energy. The enhancement seen in OD corresponds well with the increase in Fermi energy, DOS, and n_H_ with doping. It is worth mentioning that the calculated electronic band structure (Figure 5a–d) is consistent with the gradual increase in $m_{S}^{*}$ with an increase in doping amount. The gradual shift of Fermi energy towards the conduction band upon doping shows improved carrier concentration and electrical conductivity.

**Figure 6:**
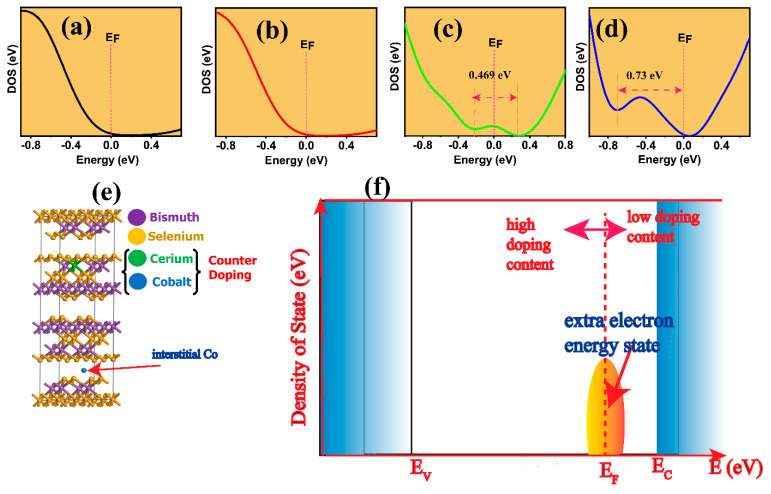
The enlarged total density of states for (**a**) BS, (**b**) BCCS-0.01, (**c**) BCCS-0.05, (**d**) BCCS-0.15, (**e**) the crystal structure of the Ce–Co counter-doped, and (**f**) the evolution of the extra electron state around the Fermi energy for low and high doing concentrations. (The blue region on the left and right denote the valence and conduction band, respectively; the arrows to the left and right from the Fermi level (E_F_) represent high and low doping position of extra energy state around the E_F_).

The simultaneous doping with transitional metals (TMs) enhances UV absorption, as the (Ce and Co) counter-doped Bi_2_Se_3_ showed improved absorption. A strong correlation exists between the absorption coefficients and average electronegativity (EN) of TM dopants, as demonstrated in Figure 6a–d. The EN difference (∆EN) between Bi (2.02) and Se (2.55) is 0.53. According to Pauling, bonding nature is described as polar covalent for (ΔEN<1.8) [42] and ionic for any other ΔEN. Thus, doping with Ce (EN → 1.12) increases the ΔEN and influences the bonding covalency of Bi-Se capacity of the pristine Bi_2_Se_3_, thus increasing the degree of delocalization of valence electrons. The self-substitution of Co (EN → 1.88) (Figure 6e) further improves the covalency of the cerium-doped bismuth selenide nanoparticulate semiconductor. It enhances the bond strength via the TM dopant’s induced electron-hole pair generation. As the EN increases, the optical density of the state (Figure 5f), via coherent improvement in the absorption coefficient, is mentioned. This is supported by the DFT, where the Co and Ce counter-doping facilitated the improvement in the electronic density of the state. This is much more feasible in the increase in the carrier concentration. Moreover, the new electron energy states (Figure 6f) around the Fermi energy serve as an electron feed to the conduction band. This new energy state extends from 0.469 eV (BCCS-0.05) to 0.73 eV (BCCS-0.15) as it swifts below the Fermi energy for higher doping content. Although the carrier concentration is enhanced, the Seebeck coefficient has not massively deteriorated. This is due to the counter-doping of the isovalent and aliovalent dopants. Unlike a single isovalent substitution of Ce in Bi_2_Se_3_, the dopant introduction only minimizes ionic impurity scattering and yields a simultaneous increase in S and σ [1].

**Figure 7:**
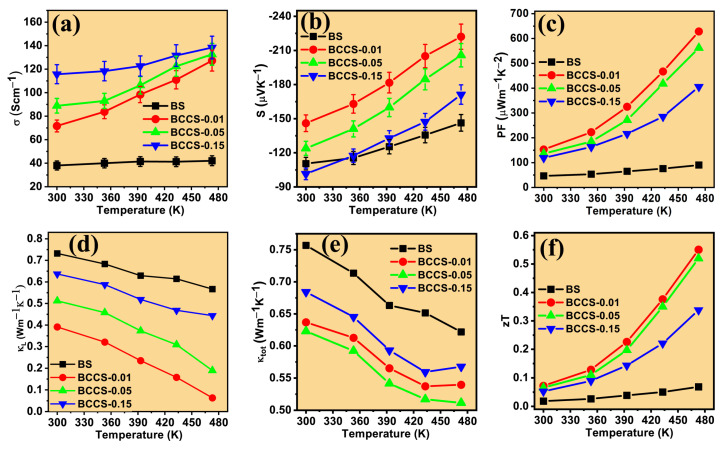
Temperature dependence of the (**a**) electrical conductivity, (**b**) Seebeck coefficient, (**c**) power factor, (**d**) lattice thermal conductivity, (**e**) total thermal conductivity, and (**f**) the figure of merit of all the synthesized BCCS samples.

The measured electrical conductivity of all the BCCS increases with doping and temperature while exhibiting a typical semiconductor behavior (Figure 7a). The increasing trend of the electrical conductivity with doping (increased n_H_) symbolizes an encroachment of the Fermi level to the conduction band. The room temperature σ of the Bi_2_Se_3_ (35 Scm^−1^) increased linearly to x = 0.15 at. % (115 Scm^−1^), representing more than three times improvement on the pristine. The monotonic increase in the electrical conductivity of all the BCCS samples with temperature signifies that the synthesized samples’ conduction types are maintained throughout the entire temperature range, with the material behaving like a degenerate semiconductor. The linear improvement in the electrical conductivity with doping greatly enhances the carrier concentration and the position of the Fermi energy from the DFT calculation (Figure 5a–d).

However, as the doping content increased to x = 0.15 at. %, a noticeable saturation in electrical conductivity is observed. This is probably due to the compensation of the isovalent and acceptor carriers. All the Co and Ce doped samples retain the n-type conduction irrespective of the doping concentrations, showing a high Seebeck coefficient compared to the pristine. Specifically, the pristine sample shows a Seebeck coefficient of −115 μV K^−1^ at room temperature, which increases by 30% (−149 μV K^−1^) at 473 K. It is interesting to note that the Seebeck coefficient increases with decreasing doping content, unlike that of the σ for the doped samples. The decrease in the Seebeck coefficient with a corresponding increase in electrical conductivity is due to the counter-doping of Co in the Ce-doped bismuth selenide materials. The doping approach favored enhancing the carrier concentration (Figure 4a—Hall measurement) for the doping concentration ranges = 0.01–0.15 at. %, thereby leading to improved electrical conductivity with a decreased Seebeck coefficient.

Increasing the doping content increases n_H_, which manifests as a corresponding increase in the σ (Figure 7a). Similarly, any doping content σ increases with temperature, which is typical of a semiconductor. This suggests that the synthesized nanoparticles are heavily doped semiconductors with Fermi energy near the conduction band. On the contrary, the Seebeck coefficient decreases with increased doping content, which also follows the usual trend of extrinsic semiconductor material with no trace of a bipolar conduction mechanism. Again, considering all the doping contents, the BCCS nanostructures exhibit a Pisarenko trend of decreasing *S* with increasing σ as a function of *n_H_*. The sensitivity of the band structure of Bi_2_Se_3_ to subtle doping can explain a significant counter-doping effect. The counter-doping approach also significantly affects all synthesized samples’ Seebeck coefficient (Figure 7b). The primary key to the remarkable improvement in transport properties of the counter-doped nanomaterials in this study is the contribution of the self-doped cobalt dopant.

The temperature dependence of the PF with different counter-doping contents is shown in Figure 7c, which clearly shows a decrease in PF with increased doping content. Considering the maximum measured temperature (473 K), a significant improvement was observed for the PF, with the PF_max_ recorded as 628 μWm^−1^ K^−2^ for x = 0.01 at. %. This is more than 12 times higher than that found for the pristine and a single Ce-doped sample reported elsewhere [1] and several other doped samples (Table S6).

The lattice ($\kappa_{L}$) and total thermal conductivity ($\kappa_{tot}$) as a function of the BCCS samples’ temperature are shown in Figure 7d and 7e, respectively. All the counter-doped samples show a lower $\kappa_{tot}$ than the pristine sample. Similarly, increasing the doping content decreases $\kappa_{tot}$ from pristine (0.62 Wm^−1^K^−1^) to x = 0.05 at. % ($\kappa_{tot}$ = 0.51 Wm^−1^K^−1^) at 473 K due to increased lattice scattering. Upon further increase in the doping amount to x = 0.15 at. %, $\kappa_{tot}$ increased ($\kappa_{tot}$ = 0.57 Wm^−1^K^−1^). It is important to understand the behavior of the $\kappa_{tot}$ by separating the $\kappa_{L}$ from the total thermal conductivity. First, $\kappa_{e}$ is directly proportional to the σ and can be estimated from the Wiedemann–Franz relation ($\kappa_{e}\sim L\sigma T)$ [43], where the Lorentz number (L) is obtained using the single parabolic band assumption [1]. Hence, using $\kappa_{tot}$ and $\kappa_{e}$, $\kappa_{L}$ is calculated ($\kappa_{tot}=\kappa_{L}+\kappa_{e}$). Incorporating the Co and Ce in the Bi_2_Se_3_ matrix effectively contributed to the scattering of the heat-carrying phonons, which reduced the $\kappa_{tot}$ to as low as 0.51 Wm^−1^K^−1^ at 473 K for x = 0.05 at. %. The overall decrease in $\kappa_{tot}\;$ can be linked to the efficient suppression of the $\kappa_{L}$ and $\kappa_{e}$ in the counter-doped samples. As doping content reaches x = 0.15 at. %, $\kappa_{tot}$ tends to rise. All the counter-doped samples were observed across the wide temperature ranges compared to the pristine, as evident by the 18% decrease from 0.62 to 0.51 Wm^−1^K^−1^ at 473 K. Thus, the larger reduction in the lattice thermal conductivity is a reason to consider that the defect structure coupled with the nano size of the particles enhances the grain boundaries and hence improves the scattering of phonons.

It is worth mentioning that the temperature dependence decreases in PF with doping coupled with the increase in $\kappa_{tot}$ for the highly doped samples renders a further increase in doping content needless. Hence, the enhanced PF and reduced $\kappa_{tot}$ for the x = 0.01 at. % sample led to a high TE performance with a zT_max_ exceeding 0.5 for x = 0.01 at. % and x = 0.05 at. % at 473 K (Figure 7f). These high zT values for the counter-doped Bi_2_Se_3_ samples originate from the enhanced electron concentration due to the band structure-manipulation-induced electronic property improvement. We have compared the results (PF_max_, $\kappa_{min}\;$, and zT_max_) in this study to some of the most recent bismuth selenide-based TE materials (Table S6). It can be seen that the counter-doping design adopted in this study offers a better approach to harnessing the full potential of bismuth selenide material for TE application. The results obtained in this study for the optimum material (Bi2−xCexCo2x3Se3,  for x=0.01 at.%) are compared with several reported studies, and it is undoubtedly shown to be among the best Bi_2_Se_3_-based [1,2,9,10,12,27,44,45,46,47,48,49].

## 4. Conclusions

In summary, we have shown Ce–Co counter-doped Bi_2−x_Ce_x_Co2x3Se_3_ (x = 0, 0.01, 0.05, and 0.15) nanoparticulate semiconductor to be an efficient TE generator within the 300 K to 473 K temperature ranges. The obtained TE performance for all the counter-doped samples showed significant improvement compared to the pristine and several reports for Bi_2_Se_3_-based TE materials. Although the case of single Ce doping in Bi_2_Se_3_ already reported showed coupled enhancement of the S and σ, the result in this study is of several orders of magnitude higher. This is because the simultaneous doping with transitional metals (TMs) enhances UV absorption, as the (Ce and Co) counter-doped Bi_2_Se_3_ showed improved optical density. A strong correlation exists between the absorption coefficients and average EN of self-doped TMs dopants via enhanced bonding covalency capacity of cerium-substituted Bi_2_Se_3_ nanoparticulate semiconductor. This leads to the enhancement of the degree of delocalization of the valence electrons and the coherent improvement in the density of state as supported by the DFT (PDOS). This counter-doping-induced enhancement in the effective density of state leads to simultaneous enhancement in the S and σ with reduced $\kappa_{tot}$ is proven by the DFT, XPS, and UV-vis. While excessive improvement in zT caused by improved carrier concentration of semiconductor materials is unusual, the synergetic effect of Ce and Co in the Bi_2_Se_3_ material surpasses the increased carrier concentration, leading to improved TE performance. Therefore, it is true to mention that the unique design of the doping eco-friendly material system accounted for the enormous potential of Bi_2_Se_3_ for application as a TE generator. The ultralow thermal conductivity is because of the efficient scattering of the phonon vibration supported by the Co impurity and small nanoparticle size (<100 nm), creating numerous scattering interfaces and boundaries.

Similarly, the Ce substitute at the Bi site promotes neutral impurities, which enhances the Seebeck coefficient without significantly deteriorating the electrical conductivity. As a result, via the synergistic effect of the Ce–Co counter doping, all the BCCS samples demonstrate better TE performance than the undoped and the single cerium doped with the highest zT = 0.55 at 473 K obtained for BCCS-0.01. This is due to the formation of a new energy state around the Fermi energy, which serves as an electron feed to the conduction band, coupled with the size of the nanomaterials, making BCCS-0.01 an optimal material for use as a TE generator. The results obtained in this study for the optimum material (Bi2−xCexCo2x3Se3) are compared with the most recent report, and it is undoubtedly shown to be among the best Bi_2_Se_3_-based TE materials already reported.

## Data Availability

The research data presented in this study are available on request from the corresponding author.

## Supplementary Materials

The following supporting information can be downloaded at: https://www.mdpi.com/article/10.3390/nano13202738/s1. Table S1: The obtained peak position, d spacing, cell parameters, and lattice volume variation in the BCCS samples; Table S2: The calculated dislocation density induced by the counter-doping for all the samples; Table S3: The stoichiometric calculation for all the synthesized samples; Table S4: Literature comparison of the binding energies and intercomponent (spin-orbit) energy separations for the measured peaks with our data; Table S5: The calculated effective mass of all the synthesized samples; Table S6: Literature comparison of the pristine and doped Bi_2_Se_3_ thermoelectric materials with our data; Figure S1: The EDX spectra of the BCCS samples (a) BS (b) BCCS-0.01 (c) BCCS-0.05 (d) BCCS-0.15; Figure S2: The EDX mapping of all the BCCS samples showing the presence of all the constituents (a) BS (b) BCCS-0.01 (c) BCCS-0.05 (d) BCCS-0.15; Figure S3: The survey spectra of all the synthesized samples (a) 100 nm fullspectra (b) Enlarged scale showing the presence of the Ce dopants.; Equation S1: The scattering mechanism in the counter-doped samples.
