# Supplementary material for: Eco-Friendly Cerium–Cobalt Counter-Doped Bi2Se3 Nanoparticulate Semiconductor: Synergistic Doping Effect for Enhanced Thermoelectric Generation
Source: Nanomaterials (Basel). 2023 Oct 10;13(20):2738. doi: 10.3390/nano13202738 (PMC10608984; doi:10.3390/nano13202738)
Supplement: Supplementary file 1 [file nanomaterials-13-02738-s001.zip › nanomaterials-2635244-supplementary.pdf]

Supporting Information

Eco-Friendly Cerium–Cobalt Counter-Doped Bi_2Se_3 Nanoparticulate Semiconductor: Synergistic Doping Effect for Enhanced Thermoelectric Generation

Jamal-Deen Musah [1,2], Siu Wing Or [1,2,*], Lingyan Kong [3], Vellaisamy A. L. Roy [4] and Chi-man Lawrence Wu [3]

[1] Department of Electrical and Electronic Engineering, The Hong Kong Polytechnic University, Hong Kong, China; jamal-deen.musah@polyu.edu.hk
[2] Hong Kong Branch of National Rail Transit Electrification and Automation Engineering Technology Research Center, Hong Kong, China
[3] Department of Materials Science and Engineering, City University of Hong Kong, Hong Kong, China; lingykong6-c@my.cityu.edu.hk (L.K.); lawrence.wu@cityu.edu.hk (C.-m.L.W.)
[4] School of Science and Technology, Hong Kong Metropolitan University, Hong Kong, China; vroy@hkmu.edu.hk
* Correspondence: eeswor@polyu.edu.hk

Table S1. The obtained peak position, d spacing, cell parameters, and lattice volume variations in the BCCS samples

Samples	d-spacing (Å)	Cell parameters(Å)		Volume (Å)^3	FWHM,β (°)
		a = b	c		
**BS**	2.0718	4.1418	28.6188	425.1601	0.1908
**BCCS-0.01**	2.0974	4.1817	27.7331	420.42862	0.4348
**BCCS-0.05**	2.0979	4.1832	27.7305	420.22979	0.6130
**BCCS-0.15**	2.0993	4.1832	27.7219	420.11504	0.7662

Table S2. The calculated dislocation density induced by the counter doping for all the samples

**Samples**	**Dislocation density ($10^{15}m^{-2}$)**
BS	0.6935
BCCS-0.01	3.501
BCCS-0.05	5.3756
BCCS-0.15	7.991

Table S3. The stoichiometric calculation for all the synthesized samples

Doping content	Bi	Ce	Co	Se	Stoichiometry
x = 0	2.017	0	0	2.983	$Bi_{2.017}Se_{2.983}$
x = 0.01	1.989	0.011	0.022	2.980	$Bi_{1.989}Ce_{0.011}Co_{0.022}$ $Se_{2.980}$
x = 0.05	1.948	0.052	0.04	2.973	$Bi_{1.948}Ce_{0.052}Co_{0.04}Se_{2.973}$
x = 0.15	1.852	0.148	0.120	2.951	$Bi_{1.852}Ce_{0.148}Co_{0.12}Se_{2.951}$

Table S4. Binding energies, intercomponent (spin-orbit) energy separations for the measured peaks in this study compared with the literature [1–3]

**Ce (III) 3d$_{5/2}$**		**Ce(III) 3d$_{3/2}$**		**Reference**
$\boldsymbol{v^o}$	$\boldsymbol{v'}$	$\boldsymbol{u^o}$	$\boldsymbol{u'}$	
881.29	884.58	899.3	903.01	**This work**
880.6	885.4	898.9	904.0	[2]
880.9	885.2	899.1	903.4	[3]
**Inter-component (spin-orbit) energy separation**				
$\Delta\ (v^o, u^o)$		$\Delta\ (v', u')$		
18.1		18.46		**This work**
18.3		18.6		[2]
18.2		18.2		[3]

Table S5. The calculated effective mass of all the synthesized samples

Samples	Effective Mass (m_s^*)
BS	0.074
BCCS-0.01	0.512
BCCS-0.05	1.269
BCCS-0.15	1.685

Table S6. Comparison of pristine and doped Bi_2Se_3 thermoelectric materials with this work.

Composition	Synthesis	Temperature (K)	PF (μW/mK)	κ_{tot}	zT	Reference
$Bi_{2-x}Ce_xCo_{2x/3}Se_3$	Solvothermal	473	628	0.539	0.55	This work
$Bi_{2-x}Sb_xSe_3$	Solvothermal	543	62	0.37	0.092	[4]
Bi_2Se_3	Solution process	473	84	0.62	0.06	[5]
$Bi_{1.9}In_{0.1}Sb_{0.067}Se_3$		473	305	0.31	0.472	
$Ce_{0.3}Bi_{1.7}Se_3$	Solvothermal	473	188	0.52	0.17	[6]
Bi_2Se_3	Solvothermal	523	152	0.83	0.096	[7]
$Bi_{1.84}Er_{0.15}Se_{2.99}$	solvothermal	480	100	0.41	0.11	[8]
$(Bi_{0.96}In_{0.04})_2Se_{2.7}Te_{0.3}$	Melting	350	1900	2.4	0.28	[9]
Bi_2Se_3	Solution	480	200	0.62	0.17	[10]
Bi_2Se_3	Bridgman method	320	410	2.1	0.063	[11]
$Cu_{0.01}Bi_{1.99}Se_3$	Melting and Hot press	473	680	0.8	0.403	[12]

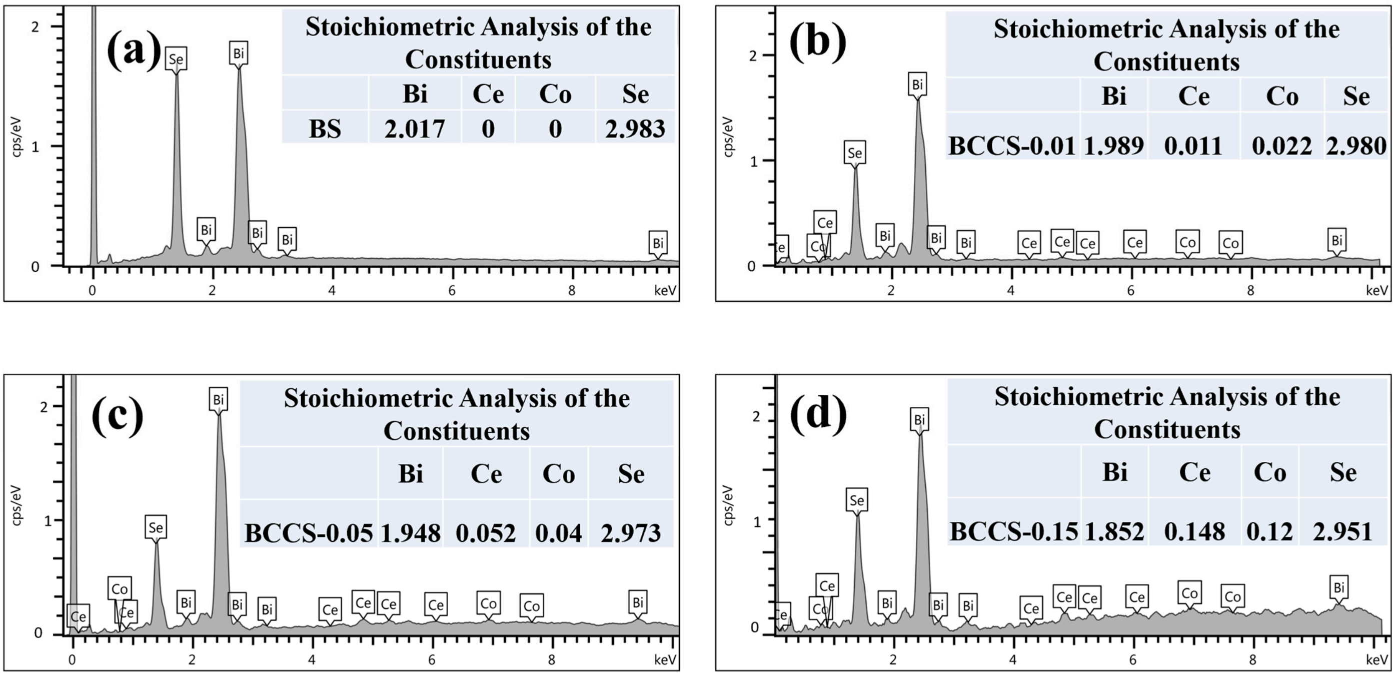

Figure S1. The EDX spectra of the BCCS samples (a)BS (b) BCCS-0.01 (c) BCCS-0.05 (d) BCCS-0.15

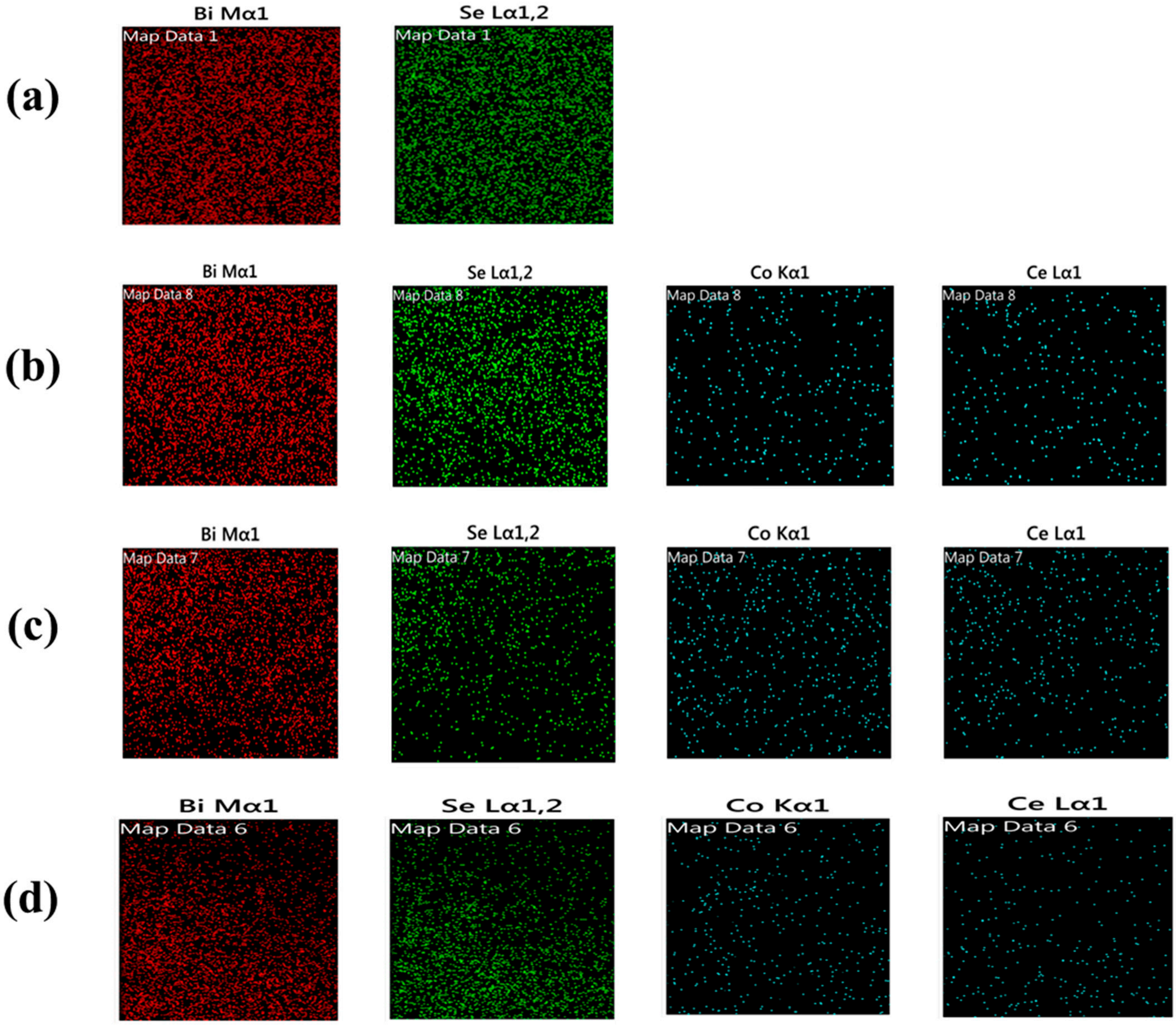

Figure S2. The EDX mapping of all the BCCS samples showing the presence of all the constitutients (a)BS (b) BCCS-0.01 (c) BCCS-0.05 (d) BCCS-0.15

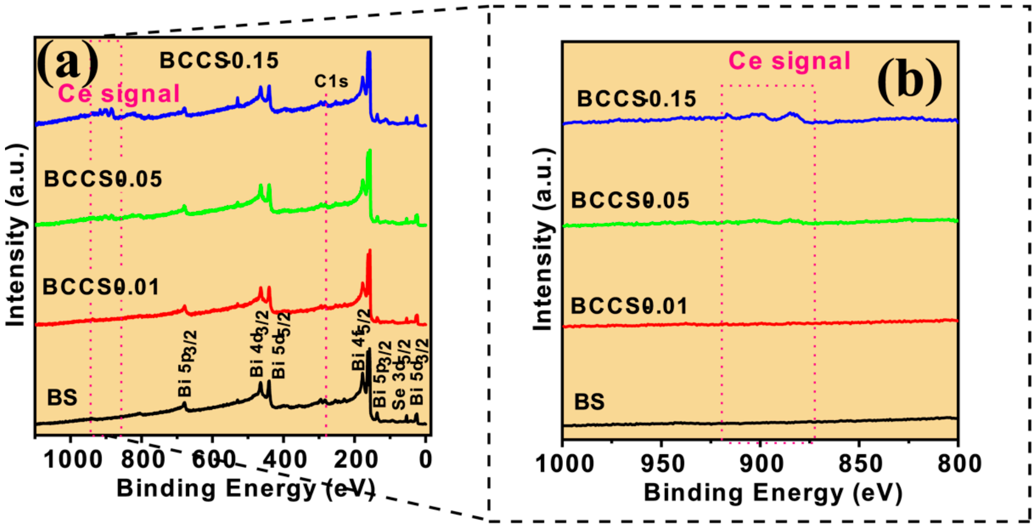

Figure S3. The Survey spectra of all synthesized samples (a) 100 nm full spectral (b) Enlarged scale showing the presence of the Ce dopants

Scattering Mechanism in the counter-doped samples

$$S = \frac{K_B}{e}\left[r + \frac{5}{2} - \frac{E_F}{KT}\right] \text{---------------------(S1)}$$

where K_B, e, E_F, T, S, and r are the Boltzmann constant, electronic charge, Fermi energy, temperature, measure Seebeck coefficient and the scattering parameter. Notably, $-1.5 \geq r \leq 1.5$

References

[1] Bêche, E.; Charvin, P.; Perarnau, D.; Abanades, S.; Flamant, G. Ce 3d XPS Investigation of Cerium Oxides and Mixed Cerium oxide ($Ce_xTi_yO_z$), *Surf. and Interface Anal*. 2008, 40, 264–267.

[2] Paparazzo, E. On the Number, Binding Energies, and Mutual Intensities of Ce3d Peaks in the XPS Analysis of Cerium Oxide Systems: A Response to Murugan et al. Superlatt. Microstruct. 2015, 85, 321. *Superlattices and Microstruct.* 2017, 105, 216–220.

[3] Wang, L.; Zhuang, L.; Xin, H.; Huang, Y.; Wang, D. Semi Quantitative Estimation of Ce^{3+}/Ce^{4+} ratio in YAG: Ce^{3+} Phosphor under Different Sintering Atmosphere. *Open J. Inorg. Chem.* **2015**, *5*, 12–18.

[4] Vijay, V.; Harish, S.; Archana, J.; Navaneethan, M. Synergistic Effect of Grain Boundaries and Phonon Engineering in Sb Substituted Bi_2Se_3 Nanostructures for Thermoelectric Applications. *J. Colloid Interface Sci.* 2022, 612 97–110.

[5] Musah, J.-D.; Linlin, L.; Guo, C.; Novitskii, A.; Ilyas, A.O.; Serhiienko, I.; Khovaylo, V.; Roy, V.A.L.; Wu, C.-M.L. Enhanced Thermoelectric Performance of Bulk Bismuth Selenide: Synergistic Effect of Indium and Antimony co-doping. *ACS Sustain. Chem. Eng.* **2022**, 10, 3862–3871.

[6] Musah, J.-D.; Yanjun, X.; Ilyas, A.M.; Novak, T.G.; Jeon, S.; Arava, C.; Novikov, S.V.; Nikulin, D.S.; Xu, W.; Liu, L.; et al. Simultaneous enhancement of Thermopower and Electrical Conductivity through Isovalent Substitution of Cerium in Bismuth Selenide Thermoelectric Materials. *ACS Appl. Mater. Interfaces* **2019**, *11*, 44026–44035.

[7] Kadel, K.; Kumari, L.; Li, W.Z.; Huang, J.Y.; Provencio, P.P. Synthesis and Thermoelectric Properties of Bi_2Se_3 Nanostructures. *Nanoscale Res Lett* **2011**, 6, 1–7.

[8] Musah, J.; Ilyas, A.M.; Novitskii, A.; Serhiienko, I.; Egbo, K.O.; Saianand, G.; Khovaylo, V.; Kwo, S.; Man, K.; Roy, V.A.L. Effective Decoupling of Seebeck Coefficient and the Electrical Conductivity through Isovalent Substitution of Erbium in Bismuth Selenide Thermoelectric Material. *J. Alloys Compd.* **2020**, *857*, 157559.

[9] Hegde, G.S.; Prabhu, A.N.; Gao, Y.H.; Kuo, Y.K.; Reddy, V.R. Potential Thermoelectric Materials of Indium and Tellurium co-doped Bismuth Selenide Single Crystals Grown by Melt Growth Technique. *J. Alloys Compd.* 2021, 866, 158814.

[10] Min, Y.; Roh, J.W.; Yang, H.; Park, M.; Kim, S. I; Hwang, S.; Lee, S.M.; Lee, K.H.; Jeong, U. Surfactant-Free Scalable Synthesis of Bi_2Te_3 and Bi_2Se_3 Nanoflakes and Enhanced Thermoelectric Properties of their Nanocomposites. *Adv. Mater.*2013, 25, 1425–1429.

[11] Dedi; Lee, P.C.; Wei, P.C.; Chen, Y.Y. Thermoelectric Characteristics of A Single-Crystalline Topological Insulator Bi_2Se_3 Nanowire. *Nanomaterials* 2021, 11, 819.

[12] Sun, G.; Qin, X.; Li, D.; Zhang, J.; Ren, B.; Zou, T.; Xin, H.; Paschen, S.B.; Yan, X. Enhanced Thermoelectric Performance of n-type Bi_2Se_3 Doped with Cu, *J. Alloys Compd.* 639, 9–14.
